# Environmental enrichment promotes resilience to neuropathic pain-induced depression and correlates with decreased excitability of the anterior cingulate cortex

**DOI:** 10.3389/fnbeh.2023.1139205

**Published:** 2023-03-16

**Authors:** Marta Falkowska, Niels R. Ntamati, Natalie E. Nevian, Thomas Nevian, Mario A. Acuña

**Affiliations:** Department of Physiology, University of Bern, Bern, Switzerland

**Keywords:** depression, neuropathic pain, anterior cingulate cortex, neuronal excitability, environmental enrichment

## Abstract

Depression is a common comorbidity of chronic pain with many patients being affected. However, efficient pharmacological treatment strategies are still lacking. Therefore, it is desirable to find additional alternative approaches. Environmental enrichment has been suggested as a method to alleviate pain-induced depression. However, the neuronal mechanisms of its beneficial effects are still elusive. The anterior cingulate cortex (ACC) plays a central role in processing pain-related negative affect and chronic pain-induced plasticity in this region correlates with depressive symptoms. We studied the consequences of different durations of environmental enrichment on pain sensitivity and chronic pain-induced depression-like behaviors in a mouse model of neuropathic pain. Furthermore, we correlated the behavioral outcomes to the activity levels of pyramidal neurons in the ACC by analyzing their electrophysiological properties *ex vivo*. We found that early exposure to an enriched environment alone was not sufficient to cause resilience against pain-induced depression-like symptoms. However, extending the enrichment after the injury prevented the development of depression and reduced mechanical hypersensitivity. On the cellular level, increased neuronal excitability was associated with the depressive phenotype that was reversed by the enrichment. Therefore, neuronal excitability in the ACC was inversely correlated to the extended enrichment-induced resilience to depression. These results suggest that the improvement of environmental factors enhanced the resilience to developing chronic pain-related depression. Additionally, we confirmed the association between increased neuronal excitability in the ACC and depression-like states. Therefore, this non-pharmacological intervention could serve as a potential treatment strategy for comorbid symptoms of chronic pain.

## 1. Introduction

Chronic pain affects around ten percent of the world’s population, varying from 19% in Europe, to 20–25% in the USA (Raffaeli and Arnaudo, 2017; Berna and Tracey, 2020), making it one of the largest health problems worldwide. Moreover, chronic pain leads to a multitude of comorbid symptoms, of which depression is the most common (Radat et al., 2013; Sheng et al., 2017), resulting in an additional deterioration in wellbeing. Therefore, deepening our understanding of the interdependence of chronic pain and depression is of high clinical relevance. The cellular and molecular mechanisms contributing to the association between depression and pain have been characterized (Humo et al., 2019). However, this understanding has not yet been translated into effective pharmacological therapies for pain-induced depression. Tricyclic antidepressants (TCAs) and serotonin/norepinephrine reuptake inhibitors (SNRIs) are among the most widely used treatments for chronic pain and pain-related depression, but a variety of side-effects and time-limiting factors constrain their use (Mico et al., 2006; Trivedi et al., 2006; Machado-Vieira et al., 2008; Gaynes et al., 2009, 2012; Moore et al., 2015). Therefore, the search for complementary approaches is of utmost importance.

Environmental enrichment (EE) relates to the optimization and enhancement of external living conditions to promote sensorimotor, cognitive stimulation and physical exercise. EE has been shown to counteract depression-like behaviors in rodents (Nithianantharajah and Hannan, 2006; Bushnell et al., 2015; Parent-Vachon and Vachon, 2018; Kimura et al., 2019, 2020, 2022; Wang et al., 2019a). In preclinical models of chronic neuropathic pain, the existence of comorbid depression-like behaviors has been demonstrated (Suzuki et al., 2007; Barthas et al., 2015; Sellmeijer et al., 2018). However, the mechanisms underlying the beneficial effects of EE on chronic pain-related depression are still not fully understood.

A large body of evidence in animal models suggests that the comorbidity of chronic pain and depression may be related to the involvement of specific brain areas (Bair et al., 2003; Doan et al., 2015; Kimura et al., 2022), particularly the hippocampus, amygdala, prefrontal cortex, and the anterior cingulate cortex (ACC) (Zhang et al., 2014; Barthas et al., 2015; Fasick et al., 2015; Yin et al., 2020; Li et al., 2021). The ACC is a key brain structure that contributes to pain processing as well emotional state integration (Apkarian et al., 2005; Shackman et al., 2011; Bushnell et al., 2013). Indeed, in depressed patients, the ACC is hyperactive (Mayberg et al., 1999; Morris et al., 2020), a condition that is also present in chronic pain patients (Apkarian et al., 2001, 2005; Bushnell et al., 2013) and preclinical pain models (Blom et al., 2014; Sellmeijer et al., 2018; Kasanetz and Nevian, 2021). Moreover, the ACC is implicated in the depressive consequences of chronic pain (Barthas et al., 2015; Koga et al., 2015), where ACC hyperactivity coincides with depression-like behavior in mice (Barthas et al., 2017; Sellmeijer et al., 2018). The local cellular and network mechanisms underlying ACC hyperactivity are based on alterations of opioidergic system, disinhibition, potentiation of excitatory synapses, and an increase in intrinsic excitability (Blom et al., 2014; Sellmeijer et al., 2018; Kasanetz and Nevian, 2021; Lee et al., 2022). A reversal of these plastic changes might therefore alleviate some of the depressive consequences in the chronic pain condition (Ru et al., 2022). However, the cellular consequences of EE on neuronal activity in the ACC that might be the basis for its therapeutic effect have not been studied yet. Furthermore, the time of enrichment relative to the development of chronic pain might be an important factor in influencing neuronal plasticity and therefore the development of depression-like symptoms.

Therefore, we characterized the effects of two types of environmental enrichment, namely, early enrichment (EEE) and extended enrichment (ExEE), on the depressive state of mice with chronic neuropathic pain, and investigated the consequences of this enrichment treatment on neuronal activity in the ACC. Using a battery of depression-like behavioral tests and *ex vivo* electrophysiology of layer 2/3 pyramidal neurons, we found that an extended protocol of environmental enrichment was sufficient to decrease pain-induced mechanical hypersensitivity and to produce resilience to depressive behaviors. In contrast, early enrichment had no beneficial effect. Moreover, we provide evidence of an inverse correlation between depression resilience and ACC neuronal hyperactivity, which supports a strong relation between neuronal activity and chronic pain-induced depression-like behaviors.

## 2. Methods

### 2.1. Animals

All experiments were conducted in accordance to the rules of the veterinary office of the canton of Bern, Switzerland. All animals (C57BL/6J male mice, Janvier Labs, France) were kept in a controlled environment (22.5°C, humidity 40–60%, lights on from 8 a.m. to 8 p.m., standard bedding material) with free access to food and water and housed in groups of four. To minimize litter effect, animals were randomly distributed in groups of four per cage, upon arrival from the Janvier Labs. All experiments were conducted under low light intensity (50–85 lux).

### 2.2. Spared nerve injury model of neuropathic pain

Neuropathic pain was induced using the Spared Nerve Injury (SNI) model as previously described (Decosterd and Woolf, 2000). Briefly, following exposure of the left sciatic nerve under 2.5% isoflurane anesthesia, the tibial and common peroneal nerve branches were ligated using silk sutures and transected while leaving the sural nerve intact. In the control condition (sham surgery), the nerve was only exposed, leaving it intact.

### 2.3. Von Frey test

Mechanical hypersensitivity was performed using the electronic version of the Von Frey test (Hogrefe et al., 2022). Mice were placed on an elevated mesh grid inside a Plexiglas cylinder and allowed to habituate for 20–30 min. Mechanical allodynia was then tested using an Electronic Von Frey aesthesiometer (EVF, IITC Life Science, CA, USA) by slowly applying pressure to the lateral plantar surface of the hind-paw with the Von Frey filament until a paw withdrawal was evoked. The pressure (in grams) for the paw withdrawal was recorded. Six pressure measures were taken per paw and an unpaired *t*-test was used as exclusion/inclusion criterion.

### 2.4. Behavioral assessment of depression-like symptoms

#### 2.4.1. Splash test (ST)

The splash test allows to evaluate loss of self-interest in animals by controlling grooming (Yalcin et al., 2011). Animals were individually placed in clean cages with a single sheet of paper towel and sprayed on the coat with 10% sucrose solution. The whole session was recorded on camera and the duration of grooming was measured during 5 min after spraying.

#### 2.4.2. Tail suspension test (TST)

Tail suspension evaluates helplessness and despair symptoms of depression-like behaviors in rodents (Vachon et al., 2013). Mice were suspended by the tail 30 cm above the surface. A two cm plastic tube was put on the tail to avoid climbing on the tail. Animals were recorded for 6 min, and the total time of immobility was measured.

#### 2.4.3. Forced swimming test (FST)

Similar to the tail suspension test, the forced swimming test evaluates depressive despair (Porsolt et al., 1977). Mice were placed into a glass cylinder (50 cm height, 30 cm diameter) filled with 20 cm of water (28°C). Immobility was assessed if the animal floated still upright in the water, without struggling or only minimally moving to prevent drowning. This test lasted for 6 min, the whole session was recorded, and the total time of immobility was calculated. Due to the high amount of stress the animals experienced this test was terminal.

Animals that escaped any of the experimental arena were excluded from the analysis. Mice were not exposed more than once to the same experimental test.

### 2.5. Housing conditions

#### 2.5.1. Enriched housing

To create an enriched environment for mice, we used large cages (26 cm × 48 cm × 20 cm), filled with 1 cm of shredded wood bedding. Mice were housed in groups of four and provided with *ad libitum* access to food and water. Each cage was equipped with a house, a wheel for voluntary physical exercising and 4–5 toys: bridges, swings, fabric hammocks, colored or white ping-pong balls, shelters, mazes, tubes, ladders, paper braids, nestlets. Non-toxic plastic toys as well as custom-made cardboard and paper objects were used (Supplementary Figure 1). Additionally, mice were randomly getting treats (small amount of sunflower and pumpkin seeds and peanuts) wrapped in paper tissues and hidden inside different types of toys to increase their interest. Cages were cleaned and disinfected once a week, and the whole setup was exchanged every cleaning session (Slater and Cao, 2015).

#### 2.5.2. Control standard housing

In the control group (SH), mice from the same batch were housed in a standard mouse cage (15 cm × 37 cm × 13 cm) (Supplementary Figure 1). No toys, tubes, or sticks were provided in the cage. However, a house and fresh single paper tissue for nesting were provided. The SH cages were cleaned and/or replaced once a week. Wellbeing of the animals was controlled daily.

### 2.6. *In vitro* patch clamp electrophysiology

Coronal sections (300 μm) of the rostral ACC were prepared using a vibratome in an ice-cold slicing solution (containing, in mM: 65 NaCl, 2.5 KCl, 25 NaHCO_3_, 1.25 NaH_2_PO_4_, 7 MgCl_2_, 0.5 CaCl_2_, 25 glucose, and 105 sucrose). After a 30-min incubation in 30°C artificial cerebrospinal fluid (aCSF, in mM: 125 NaCl, 2.5 KCl, 25 NaHCO_3_, 1.25 NaH_2_PO_4_, 1 MgCl_2_, 2 CaCl_2_, and 25 glucose), and at least 30 additional minutes of recovery at room temperature, ACC slices were transferred to a recording chamber constantly superfused with 30°C aCSF (2 ml/min). All solutions used were constantly bubbled with a 95% O_2_ and 5% CO_2_ gas mixture. Borosilicate glass pipettes were pulled at a resistance of 4–7 MΩ and filled with an internal solution containing, in mM: 130 K-gluconate, 5 KCl, 10 Na-phosphocreatine, 4 Mg-ATP, 0.3 Na-GTP, 10 HEPES, and 2 mg/ml biocytin (for morphological characterization, see below). Layer 2/3 neurons were visually identified under infrared LED illumination (Thorlabs, NJ, USA) through an IR CCD camera mounted on a Leica DM LFSA microscope (IL, USA). Whole-cell current clamp recordings were filtered at 5 kHz and amplified, then digitized at 10 kHz and stored on hard disk. All data were acquired and analyzed using custom-made MATLAB (MathWorks) codes. Intrinsic neuronal excitability was assessed by quantifying the action potential firing frequency response to ten progressively increasing square current injections (from −300 pA to +600 pA). From this input-output relationship, the action potential (AP) threshold was estimated as the minimum current required to elicit a single action potential derived from a spline function from the input/output curve, and the input resistance *R*_*in*_ was calculated from the hyperpolarization △*V* evoked at −300 pA (*I*), according to the relationship *R*_*in*_ = △*V*/*I*.

#### 2.6.1. Morphological characterization of L2/3 pyramidal neurons

After patch-clamp electrophysiology recordings were terminated, we characterized the morphology of the recorded neurons, as previously described (Hogrefe et al., 2022). Briefly, brain slices were fixed in 4% PFA at 4°C overnight, and permeabilized in PBS containing 2% Triton X-100 for 1 h. The slices were incubated with streptavidin-conjugated Alexa-488 (1:200; Invitrogen) in PBS containing 1% Triton X-100, then washed 3x with PBS, before embedding them in Mowiol on microscopy slides. Fluorescently labeled slices were imaged using a confocal microscope (Leica Microsystems, SP8) equipped with a white-light laser and two GaAsP-detectors (HyD). Imaging was performed with a 20 × objective (Leica Microsystems, HC PL APO, 20 ×, NA 0.75 IMM CORR CS2). The region containing the labeled pyramidal neuron was imaged in the tile-scan mode from the midline to L6. The morphological features of pyramidal neurons were characterized manually in ImageJ by full apical dendrite extension toward layer 1, and the presence of basal dendrites. Neurons that had cut dendrites or morphological similarities to interneurons were excluded from the analysis. A total of 122 neurons passed the selection criteria for pyramidal neuron classification. No more than four neurons per slice were recorded. Per animal we obtained 3–4 number of slices. A total of 16 animals were used (SNI-ExEE, *N* = 3; SNI-SH, *N* = 6, sham-ExEE, *N* = 3; sham-SH = 4).

### 2.7. Correlation of depression resilience and neuronal excitability

We analyzed the association between neuronal excitability and resilience to depression within a group of animals (*N* = 16, Figure 4H). We z-scored the behavioral response to the splash test, FST and TST and neuronal excitability indicators such as AP threshold, and input resistance across all experimental groups. The z-scoring was aimed to normalize behavioral and excitability values across tests. For the FST and TST, high values of time spend immobile indicate depression, whereas in the splash test, lower grooming time indicates depression. Therefore, resilience was calculated as z-score values for splash test, and the additive inverse z-score values for the FST and TST. Same procedure was conducted for the electrophysiological properties, across groups. Low AP threshold corresponds to high excitability, and high input resistance relate also to high excitability. Therefore, Neuronal excitability was calculated as the z-score values of input resistance and the additive inverse z-score values of AP threshold. We then averaged the values of excitability across cells and obtained a grand average per animal. This allowed us to match behavioral responses and neuronal excitability at the same scale. Finally, the values of depression resilience vs. neuronal excitability per animal were plotted, and a regression line was calculated to obtain the correlation coefficient.

### 2.8. Statistics

Statistical analyses were carried out using MATLAB and GraphPad Prism 9.3.1. The time course for sensory measures considering contra- and ipsilateral paws were analyzed by 2-way repeated measures analysis of variance (ANOVA) followed by the Tukey *post-hoc* test for multiple comparisons. All other behavioral tests outcomes were analyzed by unpaired *t*-test, where only two groups where compared, or two-way ANOVA followed by *post-hoc* tests for multiple comparisons. All data are expressed and plotted as mean ± SEM. Significance threshold was set at *p* < 0.05.

### 2.9. Code availability

All codes for the electrophysiological analysis performed in this study were written are publicly available at https://github.com/marioaacuna/in-vitro-ephys.

## 3. Results

### 3.1. Early exposure to environmental enrichment alone does not alleviate depression-like symptoms in SNI animals

**FIGURE 1:**
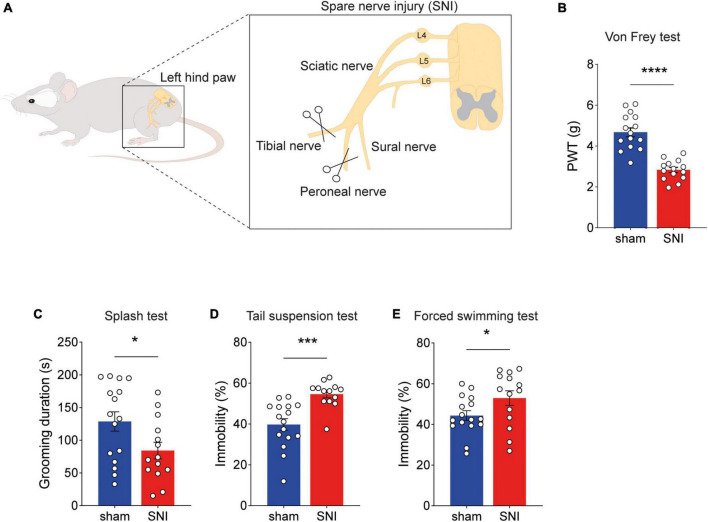
Depression-like behaviors develop after chronic neuropathic pain. **(A)** Schematics of sciatic nerve injury (SNI) model of neuropathic pain. **(B)** Paw withdrawal threshold (PWT) in SNI and sham animals measured by the electronic von Frey test. Hypersensitivity in SNI animals significantly increased 8 weeks post-surgery (****p < 0.0001). **(C–E)** Behavioral assessment of depression-like symptoms. SNI animals displayed significant reduction in grooming duration in the splash test [**(C)**, *t* = 2.250, *df* = 28, *p = 0.0325], increased immobility time in the tail suspension test [**(D)**, *t* = 4.167, *df* = 27, ***p = 0.0003], and in the forced swimming test [**(E)**, *t* = 2.052, *df* = 28, *p = 0.0496]. *n* = 13–16 mice per group. Data presented as mean ± SEM.

Long-lasting neuropathic pain has been associated with a progressive development of depression-like behaviors (Sellmeijer et al., 2018). We therefore evaluated the depressive state of chronic neuropathic pain animals housed in standard conditions by three well-established protocols, namely the sucrose splash test (ST), the tail suspension test (TST), and the forced swimming test (FST) (Kremer et al., 2021). We found that 8 weeks after inducing neuropathic pain by the spared nerve injury (SNI) model (Figure 1A), SNI animals showed mechanical hypersensitivity (Figure 1B). Moreover, we found behavioral symptoms of depression, such as lower cumulative duration of grooming in SNI animals (Figure 1C), increased immobility in the TST (Figure 1D) and FST (Figure 1E). These results suggest that chronic neuropathic pain leads to depression-like symptoms.

**FIGURE 2:**
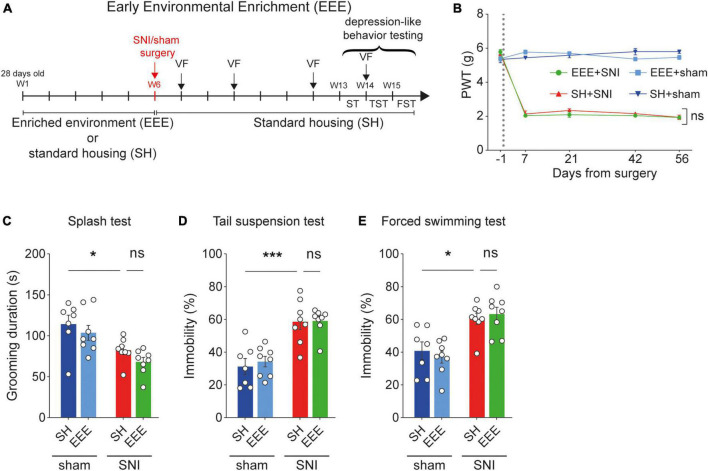
Early environmental enrichment (EEE) fails to protect against pain-induced hypersensitivity and depression-like behaviors. **(A)** Timeline of the EEE protocol. **(B)** Paw withdrawal threshold (PWT) values measured using von Frey test across the duration of experiment. EEE protocol caused no significant changes in PWT between distinct housing groups in the neuropathic pain group. For days +7, +21, +42, and +56 after surgery, two-way ANOVA, for the interaction housing and surgery *F*_(1,27)_ = 3.193, *p* = 0.0852, *F*_(1,27)_ = 2.206, *p* = 0.1490, *F*_(1,27)_ = 1.888, *p* = 0.1808, *F*_(1,27)_ = 2.061, *p* = 0.1626, respectively. *Post-hoc* Tukey’s multiple comparisons correction, ns, *p* > 0.05; *n* = 7–8 mice per condition. **(C–E)** Behavioral measurements of depression-like behaviors are not recovered by the EEE protocol. **(C)** Splash test. Two-way ANOVA, surgery effect (SNI vs. sham) *F*_(1,27)_ = 18.25, *p* = 0.0002; housing effect (SH vs. EEE) *F*_(1,27)_ = 2.474, *p* = 0.1274. *Post-hoc* Tukey’s multiple comparisons correction, *p < 0.05. **(D)** Tail suspension test. Two-way ANOVA, surgery effect (SNI vs. sham) *F*_(1,27)_ = 43.73, *p* < 0.0001; housing effect (SH vs. EEE) *F*_(1,27)_ = 0.2001, *p* = 0.6582. *Post-hoc* Tukey’s multiple comparisons correction, ***p < 0.001. **(E)** Forced swimming test. Two-way ANOVA, surgery effect (SNI vs. sham) *F*_(1,27)_ = 30.35, *p* < 0.0001; housing effect (SH vs. EEE) *F*_(1,27)_ = 0.01528, *p* = 0.9025. *Post-hoc* Tukey’s multiple comparisons correction, *p = 0.0144. *N* = 7–8 mice per condition. Data presented as mean ± SEM.

Next, we investigated whether early exposure to environmental enrichment (EEE, Figure 2A and Supplementary Figure 1A) could have protective effects against hypersensitivity and pain-related depression-like symptoms in SNI animals. We exposed 31 mice (four-week-old) to enriched environments (*n* = 15) or standard housing (*n* = 16) for 6 weeks, after which animals were separated into SNI (*n* = 8) and sham (*n* = 7–8) groups. After surgery, EEE animals were placed in standard cages for another 6 weeks. We found that the EEE protocol failed to protect against neuropathic pain-induced mechanical hypersensitivity (Figure 2B). Also, the EEE protocol did not prevent the development of depression-like behaviors in neuropathic pain animals (Figures 2C–E). These results suggest that early exposure to an enriched environment prior to the induction of neuropathic pain is not sufficient to develop resilience to pain-related mechanical sensitivity and pain-induced depressive symptoms.

### 3.2. Extended exposure to environmental enrichment protects against chronic pain-induced mechanical hypersensitivity and depression-like symptoms

**FIGURE 3:**
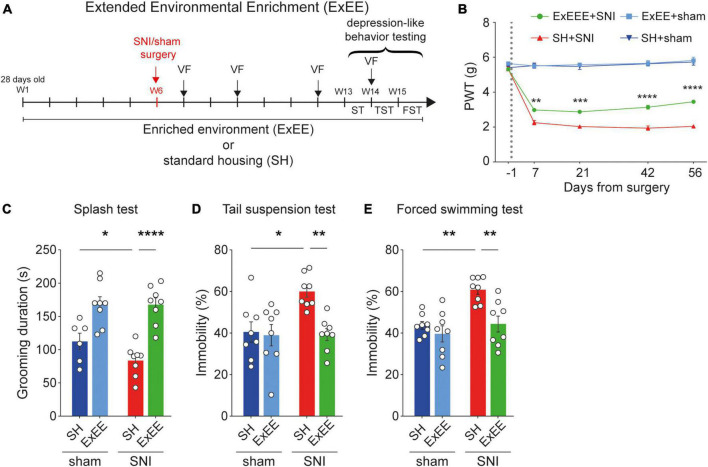
Extended environmental enrichment reduces mechanical hypersensitivity and promotes resilience to depression. **(A)** Timeline of the ExEE protocol. **(B)** Paw withdrawal threshold (PWT) values measured using von Frey test across the duration of experiment. ExEE protocol protected against the full development of mechanical hypersensitivity in SNI animals. For days +7, +21, +42, and +56 after surgery, two-way ANOVA, for the interaction housing and surgery *F*_(1,28)_ = 11.29, *p* = 0.0023, *F*_(1,28)_ = 7.5773, *p* = 0.0103, *F*_(1,28)_ = 21.76, *p* < 0.0001, *F*_(1,28)_ = 26.33, *p* < 0.0001, respectively. *Post-hoc* Tukey’s multiple comparisons correction, **p < 0.01, ***p < 0.001, ***p < 0.0001, between SH-SNI and ExEE-SNI; *n* = 7–8 mice per condition. **(C–E)** ExEE protects against depression-like behaviors. **(C)** Splash test. Two-way ANOVA, surgery effect (SNI vs. sham) *F*_(1,26)_ = 43.00, *p* < 0.0001; housing effect (SH vs. ExEE) *F*_(1,26)_ = 1.877, *p* = 0.1824. *Post-hoc* Benjamini, Krieger, and Yekutieli multiple comparisons correction, ****p < 0.0001, *p < 0.05. **(D)** Tail suspension test. Two-way ANOVA, surgery effect (SNI vs. sham) *F*_(1,28)_ = 7.504, *p* = 0.0061; housing effect (SH vs. ExEE) *F*_(1,28)_ = 5.915, *p* = 0.0217. *Post-hoc* Tukey’s multiple comparisons correction, **p < 0.01, *p < 0.05. **(E)** Forced swimming test. Two-way ANOVA, surgery effect (SNI vs. sham) *F*_(1,28)_ = 12.69, *p* = 0.0013; housing effect (SH vs. EEE) *F*_(1,28)_ = 0.0023, *p* = 0.9025. *Post-hoc* Tukey’s multiple comparisons correction, **p < 0.01. Data presented as mean ± SEM.

Given the lack of protective effect of the early enrichment housing prior to surgery, we designed an extended version of the enrichment protocol, where the animals where continuously exposed to the enriched environment both before and after surgery. For this protocol, a different cohort of animals (*n* = 32) was exposed to the extended environmental enrichment protocol (*n* = 16, ExEE, Figure 3A). Mice experienced again 6 weeks of enrichment before the SNI or sham surgery. However, they were kept in their respective enriched housing conditions also after surgery (ExEE sham, *n* = 8; ExEE SNI, *n* = 8). Housing control littermates were kept in standard housing (SH) conditions throughout the duration of the protocol (SH sham, *n* = 8; SH SNI, *n* = 8). We found that the ExEE protocol prevented the full development of mechanical hypersensitivity in SNI animals already from post-operative day +7 (Figure 3B), with a significant increase of the paw withdrawal threshold over time (Supplementary Figure 2A), indicating a temporal effect of the enrichment on mechanical hypersensitivity. Moreover, the ExEE protocol in SNI animals fully protected against the development of depression-like behaviors as observed in SNI mice housed in standard conditions (Figures 3C–E). These results suggest that extended enriched housing attenuates neuropathic pain-induced mechanical hypersensitivity and induces resilience to depression in chronic neuropathic pain animals.

### 3.3. Resilience to depression is associated with decreased neuronal activity in the ACC

**FIGURE 4:**
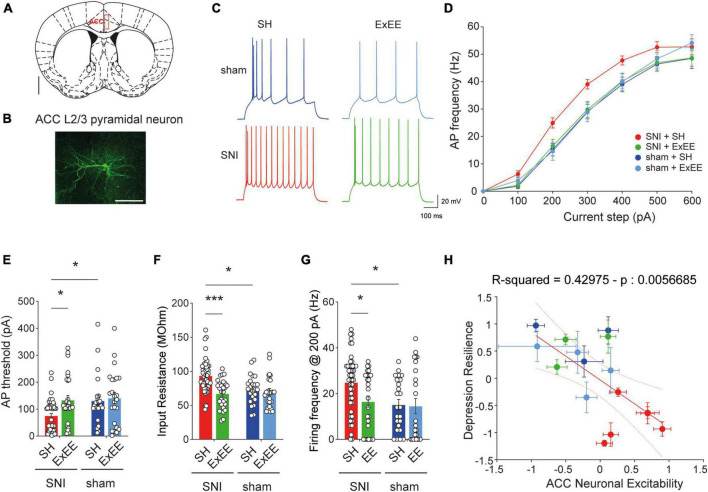
Extended environmental enrichment (ExEE) restores neuronal excitability in ACC L2/3 pyramidal neurons. **(A)** Schematic representation of ACC location according to the mouse brain atlas. Scale bar, 1 mm. **(B)** Representative reconstruction of ACC L2/3 pyramidal neuron after patch clamp electrophysiological characterization. Scale bar, 100 μm. **(C)** Representative action potential (AP) firing upon 200 pA current pulse for cells from animals that underwent different housing conditions and surgeries. Scale bars, 20 mV and 100 ms. **(D)** Increased gain of input-output curve in SNI animals housed in standard conditions is reversed by the ExEE. **(E)** AP firing threshold quantification. Two-way ANOVA, surgery effect (SNI vs. sham) *F*_(1, 119)_ = 4.847, *p* = 0.0296; housing effect (SH vs. ExEE) *F*_(1,119)_ = 6.480, *p* = 0.0122. *Post-hoc* Holm-Šídák’s multiple comparisons correction, *p < 0.05. **(F)** Input resistance. Two-way ANOVA, surgery effect (SNI vs. sham) *F*_(1,119)_ = 5.191, *p* = 0.0245; housing effect (SH vs. ExEE) *F*_(1,119)_ = 16.23, *p* < 0.0001. *Post-hoc* Holm-Šídák’s multiple comparisons correction, ***p < 0.001, *p < 0.05. **(G)** Action potential firing frequency at 200 pA. Two-way ANOVA, surgery effect (SNI vs. sham) *F*_(1,119)_ = 6.298, *p* = 0.0134; housing effect (SH vs. ExEE) *F*_(1,119)_ = 4.542, *p* = 0.0351. *Post-hoc* Holm-Šídák’s multiple comparisons correction, *p < 0.05. **(H)** Significant inverse correlation between depression-like behaviors and ACC neuronal excitability. Each point represents one animal with Y axis depicting the normalized behavioral responses and X axis depicting normalized neuronal excitability. Depression resilience was determined as normalized values for each of the three depression-like tests. Neuronal excitability indicates normalized values of AP threshold and input resistance per animal. Data represent mean ± SEM.

Several lines of evidence suggest that neuropathic pain leads to hyperexcitability of neurons in the ACC (Blom et al., 2014; Sellmeijer et al., 2018; Kasanetz and Nevian, 2021; Lee et al., 2022). Moreover, the depressive state is thought to depend particularly on cellular changes in layer 2/3 pyramidal neurons (Sellmeijer et al., 2018). However, it is elusive whether environmental enrichment restores the excitability phenotype. Consequently, we investigated the potential changes in neuronal activity of layer 2/3 pyramidal neurons (Figures 4A, B) induced by the ExEE protocol compared to the standard housing condition, by means of whole-cell patch-clamp electrophysiology (Figures 4C–G). We found that the ExEE protocol in SNI animals restored the input-output curve (Figures 4C, D, G), action potential (AP) threshold (Figure 4G), and input resistance (Ri, Figure 4F) to sham levels, while resting membrane potential, membrane time constant, AP amplitude and AP shape were not different in any condition (Supplementary Figures 3A–G). *Post-hoc* comparison of the electrophysiological parameters of individual animals to their depression-like state yielded a significant inverse correlation between the degree of resilience to depression and neuronal excitability (Figure 4H). These data therefore highlight the association between ACC neuronal excitability and the depressive state of the animals.

## 4. Discussion

We found that an extended environmental enrichment that starts at post-natal day 28 and continues for 18 weeks can reduce the mechanical pain phenotype and importantly unmask resilience to chronic pain-induced depression-like behaviors. Additionally, we were able to associate this resilience to depression to the excitability state of L2/3 pyramidal neurons in the ACC, a brain area within the pain matrix involved in affective and emotional processing. ACC neurons of neuropathic pain animals housed in standard conditions showed hyperexcitability as manifested by an increase in input resistance and a decrease of AP firing threshold, all of which could be reversed by environmental enrichment, suggesting a relation between cellular plastic changes and the presence of pain-induced depression-like behaviors. Our results therefore stress the importance of environmental enrichment as a potential alternative strategy to prevent the development of depressive disorders in chronic pain and indicate that the regulation of neuronal activity in the ACC might be the basis of such beneficial effects. Additionally, we present data supporting the central role of the ACC in chronic pain-induced mood disorders. Moreover, we provide novel evidence for a physical intervention that modulates the activity of ACC neurons and results in resilience to depression in neuropathic pain animals.

In this study, we showed that an early exposure to an enriched environment limited to the time before the induction of neuropathic pain, failed to reduce neuropathic pain-induced mechanical hypersensitivity or behavioral symptoms of depression. However, a combination of pre-and post-operative environmental enrichment successfully prevented both the full development of allodynia and depression-like behaviors in chronic neuropathic pain mice. In contrast to previous studies (Wang et al., 2019b), we observed that our protocol induced an early protection from mechanical hypersensitivity in neuropathic animals, already from day +7 after surgery. This difference might arise from the timing of placing the animals into the enriched environment, arguing in favor of a continuous exposure to environmental enrichment. Our study therefore provides evidence that the early onset of environmental enrichment, together with an uninterrupted continuation of the exposure, prevents the full development of allodynia, characteristic of neuropathic pain states, even at early stages. This then suggests that a timely applied enrichment may determine the power of the treatment’s effectiveness (Low et al., 2013).

Our electrophysiological data in the neuropathic pain condition show that environmental enrichment induces homeostatic plastic changes which decrease cellular excitability to balance the network architecture. In contrast, environmental enrichment has been associated with increased excitability in the healthy brain. In the hippocampus, this leads to enhanced learning and memory (Rampon et al., 2000a; Hullinger et al., 2015). In the visual cortex, increased excitability promotes ocular dominance plasticity (Kalogeraki et al., 2017) and in the somatosensory cortex, it improves sensory discrimination (Alwis and Rajan, 2013). However, in the diseased brain, EE reverses the altered state observed in neurodegenerative diseases (van Praag et al., 2000; Nithianantharajah and Hannan, 2006). Intriguingly, a recent report found that EE changes the transcriptional signature of cortical neurons leading to a dampening of overexcitability, thereby promoting cognitive performance in mice (Barker et al., 2021). This, together with previous reports (Rampon et al., 2000b; Nithianantharajah et al., 2004), suggests that EE induces transcriptional, and translational changes that overall regulate excitability of neurons depending on the initial state of the brain. Therefore, EE might restore cellular changes that control excitability, so that the neuronal network function is improved, protecting the brain against deleterious effects of insults, including chronic pain. Indeed, chronic pain leads to changes in transcriptional patterns (Su et al., 2021; Wang et al., 2021; Dai et al., 2022), together with epigenetic changes (Mauceri, 2022), along the pain neuraxis that could contribute to alterations in excitability and network activity, all of which could be potentially reversed by EE. Overall, EE is a promising approach for restoring neuronal excitability in rodents and may have potential applications for the treatment of neurological disorders in humans (Kimura et al., 2022). However, more research is needed to fully understand the mechanisms underlying these effects and to determine the potential of environmental enrichment as a therapeutic intervention.

Recent evidence suggest that voluntary exercise could be sufficient to prevent the full expression of mechanical hypersensitivity and depression-like behaviors in rodents (Grace et al., 2016; Eldomiaty et al., 2017; Pagliusi et al., 2020; Ferrarini et al., 2021) and humans (Marshall et al., 2017; Izquierdo-Alventosa et al., 2020). However, we could not differentiate between the contribution of increased exercise and the cognitive challenges through sensory stimulation in our ExEE housing protocol. It is therefore tempting to speculate that exercise alone might have protective effects against pain-induced depression, by also reducing ACC hyperactivity, however, this needs to be further characterized.

Our study was limited to the evaluation only in male mice. It has been shown that the development of chronic pain in female mice diverges from that of male through specific mechanisms (Melchior et al., 2016; Mapplebeck et al., 2018). Indeed, sexual dimorphism in chronic pain has been observed in transcriptomic profiles in the ACC and in the medial prefrontal cortex (Dai et al., 2022), which may lead to differential susceptibilities and re-configurations by EE. It is therefore imperative to evaluate in future research the mechanisms of EE-promoted resilience to neuropathic pain-induced comorbid symptoms in female mice (Girbovan and Plamondon, 2013).

Taken together, our results provide evidence of a non-pharmacological treatment against mechanical hypersensitivity and depression-like behaviors. Moreover, we confirm the association between dampened neuronal excitability in the ACC and resilience to depression in chronic neuropathic pain, suggesting a possible therapeutic intervention *via* modulation of activity in the ACC (Drobisz and Damborska, 2019; Cole et al., 2022) for treatment of depression in chronic pain patients.

## Data availability statement

The original contributions presented in this study are included in the article/Supplementary material, further inquiries can be directed to the corresponding author.

## Ethics statement

This animal study was reviewed and approved by the Veterinary Office of the Canton of Bern, Switzerland.

## Author contributions

MF, TN, and MA designed the experiments. MF performed the behavioral experiments. NRN performed the electrophysiological experiments. NEN performed the biocytin neuronal reconstructions. MF and MA analyzed data. MA wrote the manuscript. All authors contributed to the article and approved the submitted version.
