# Supplementary material for: Environmental enrichment promotes resilience to neuropathic pain-induced depression and correlates with decreased excitability of the anterior cingulate cortex
Source: Front Behav Neurosci. 2023 Mar 16;17:1139205. doi: 10.3389/fnbeh.2023.1139205 (PMC10060563; doi:10.3389/fnbeh.2023.1139205)
Supplement: Supplementary file 1 [file Data_Sheet_1.docx]

**Supplementary Material**

**
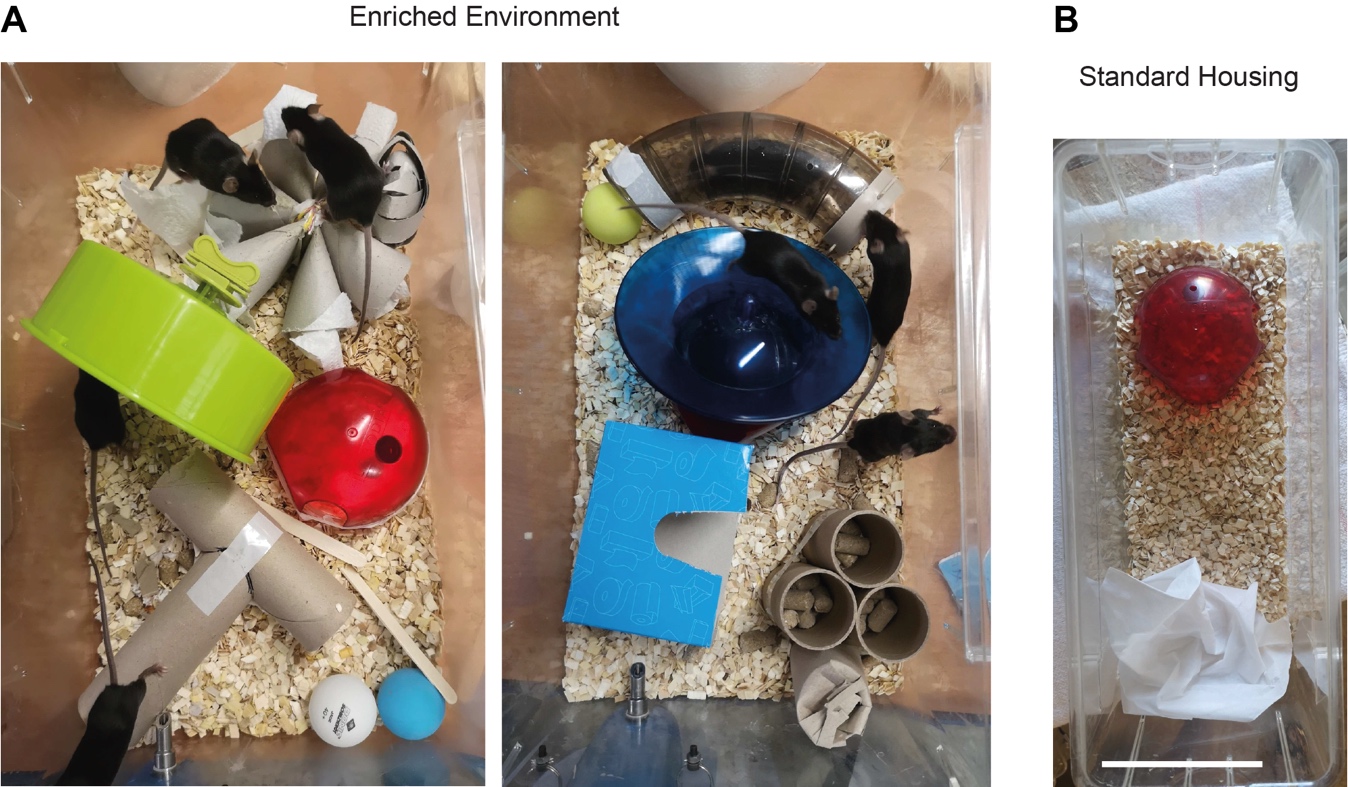
**

**Figure S1**

Examples of EN cages. Diverse materials and toys were used in numerous setup combinations in order to stimulate exploratory behavior and learning in mice from EN groups. Right: the equipment of a standard housing cage. Scale bar 10 cm.

**
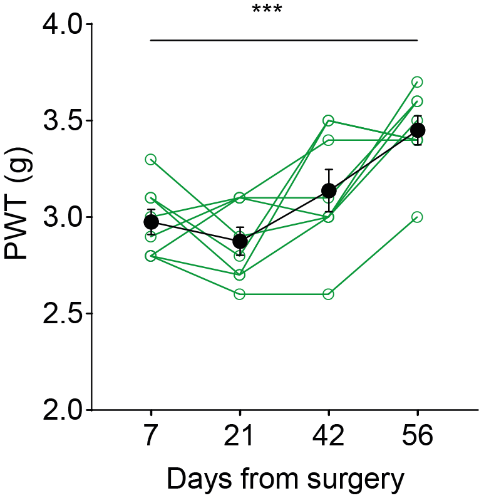
**

**Figure S2**

Significant increase in postsurgical PWT of neuropathic animals housed in enriched environment. Data taken from Fig. 3B. One-way ANOVA, F (3, 21) = 13.06, p < 0.0001, day 7 x day 56: p = 0.0005, n = 8.


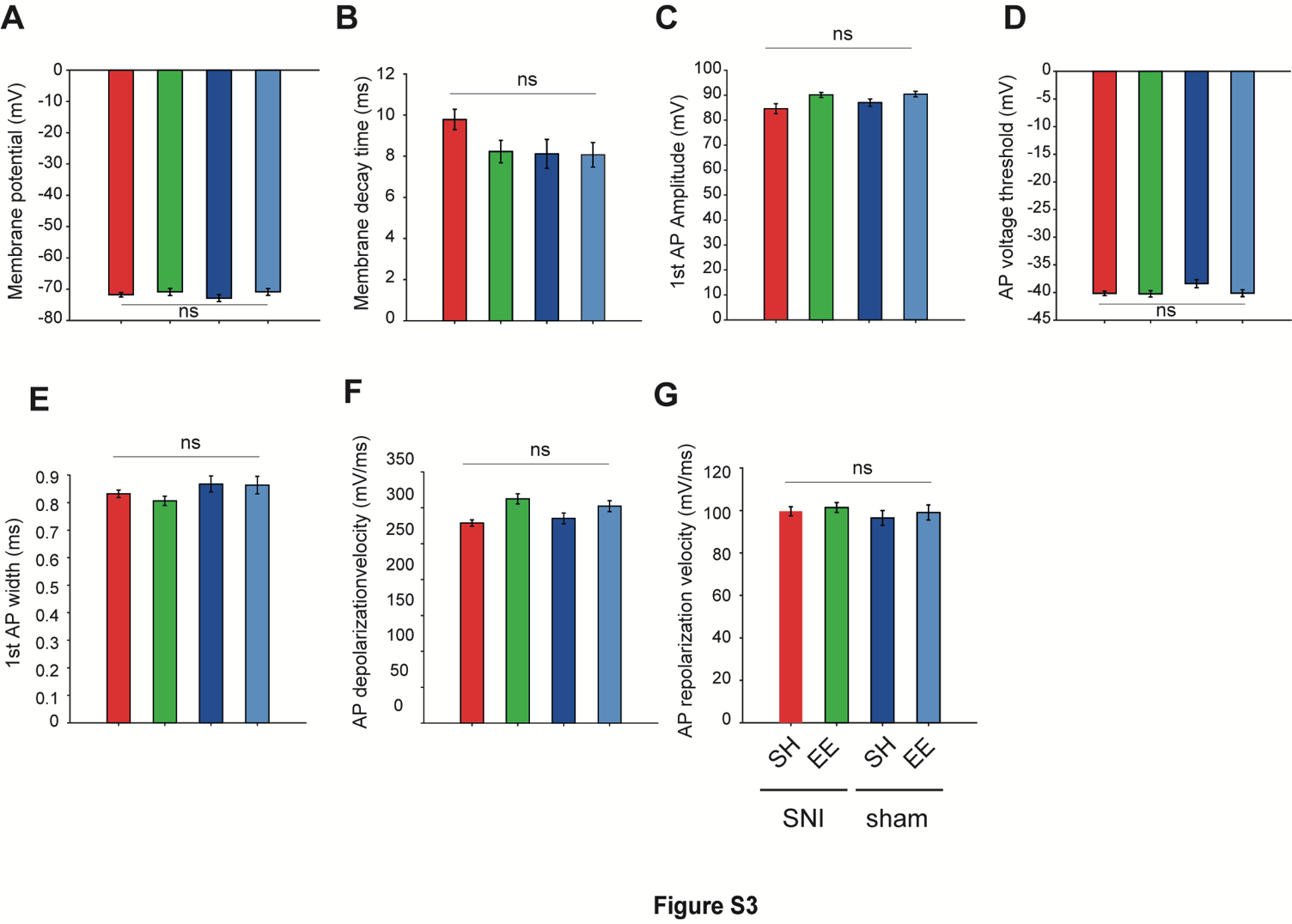


**Figure S3**

Unaltered electrophysiological properties. **(A)** Resting membrane potential. Two-way ANOVA, surgery effect (SNI vs sham) F(1,119) = 3.4409, p = 0.067; housing effect (SH vs ExEE) F(1,119) = 0.8821, p = 0.349. **(B)** Membrane decay time. Two-way ANOVA, surgery effect (SNI vs sham) F(1,119) = 1.0898, p = 0.2987; housing effect (SH vs ExEE) F(1,119) = 3.34, p = 0.0698. **(C)** First action potential (AP) amplitude measured from voltage threshold. Two-way ANOVA, surgery effect (SNI vs sham) F(1,119) = 1.555, p = 0.2149; housing effect (SH vs ExEE) F(1,119) = 0.6363, p =0.427. **(D)** Action potential firing threshold, obtained from dV/dt >= 5mV/ms. Two-way ANOVA, surgery effect (SNI vs sham) F(1,119) = 2.647, p = 0.1064; housing effect (SH vs ExEE) F(1,119) = 2.4706, p = 0.1187. **(E)** First AP width. Two-way ANOVA, surgery effect (SNI vs sham) F(1,119) = 0.1621, p = 0.6879; housing effect (SH vs ExEE) F(1,119) = 3.35, p = 0.0697. **(F)** AP depolarization velocity. Two-way ANOVA, surgery effect (SNI vs sham) F(1,119) = 19.079, p = 0.0003; housing effect (SH vs ExEE) F(1,119) = 0.5029, p = 0.4796. **(G)** AP repolarization velocity. Two-way ANOVA, surgery effect (SNI vs sham) F(1,119) = 0.2301, p = 0.6324; housing effect (SH vs ExEE) F(1,119) = 0.4708, p = 0.4940.
