# Supplementary material for: Environmental enrichment promotes resilience to neuropathic pain-induced depression and correlates with decreased excitability of the anterior cingulate cortex
Source: Front Behav Neurosci. 2023 Mar 16;17:1139205. doi: 10.3389/fnbeh.2023.1139205 (PMC10060563; doi:10.3389/fnbeh.2023.1139205)
Supplement: Supplementary file 2 [file Table_1.DOCX]

**Supplementary Table 1. Mean and Standard Error of the Mean (SEM) values for main Figures.**

Figure. 1

| Von Frey N = 14 | SNI | 2.828 (+-0.1301) |
| --- | --- | --- |
| N = 16 | Sham | 4.668 (+- 0.2144) |
| Splash test (grooming duration) (s) N = 14 | SNI | 84.14 (+-12.73) |
| N = 16 | sham | 128.8 (+-14.81) |
| Tail Suspension test (immobility %) N = 13 | SNI | 54.57 (+-1.783) |
| N = 16 | Sham | 39.76 (+-2.852) |
| Forced swimming test (immobility %) N = 14 | SNI | 52.95 (+-3.580) |
| N = 16 | sham | 44.33 (+-2.376) |

Figure. 2

| Method | Housing | Condition | Recorded value – mean (SEM) |
| --- | --- | --- | --- |
| Von Frey (week 1) N = 8 | EEE | SNI | 2.038 (+- 0.073) |
| N = 8 | EEE | Sham | 5.775 (+-0.124) |
| N = 8 | Standard (EEE) | SNI | 2.138 (+-0.176) |
| N = 7 | Standard (EEE) | Sham | 5.443 (+- 0.061) |
| Von Frey (day 56) N = 8 | EEE | SNI | 1.925 (+-0.082) |
| N = 8 | EEE | Sham | 5.463 (+- 0.122) |
| N = 8 | Standard (EEE) | SNI | 1.938 (+- 0.131) |
| N = 7 | Standard (EEE) | Sham | 5.8 (+- 0.109) |
| Splash test – grooming duration (s) N = 8 | EEE | SNI | 86.125 (+- 5.380) |
| N = 8 | EEE | Sham | 103.625 (+-9.106) |
| N = 8 | Standard (EEE) | SNI | 82.375 (+- 5.199) |
| N = 7 | Standard (EEE) | Sham | 114.143 (+-11.072) |
| Tail Suspension Test N = 8 | EEE | SNI | 59.063 (+- 2.817) |
| N = 8 | EEE | Sham | 34.201 (+-3.203) |
| N = 8 | Standard (EEE) | SNI | 58.576 (+-4.684) |
| N = 7 | Standard (EEE) | Sham | 31.151 (+-4.908) |
| Forced Swimming Test N = 8 | EEE | SNI | 63.229 (+- 4.204) |
| N = 8 | EEE | Sham | 36.910 (+-3.640) |
| N = 8 | Standard (EEE) | SNI | 60.417 (+-3.402) |
| N = 7 | Standard (EEE) | Sham | 40.754 (+-5.461) |

Figure. 3

| Method | Housing | Condition | Recorded value – mean (SEM) |
| --- | --- | --- | --- |
| Von Frey (week 1) N = 8 | ExEE | SNI | 2.975 (+- 0.065) |
| N = 8 | ExEE | Sham | 5.5 (+- 0.08) |
| N = 8 | Standard (ExEE) | SNI | 2.25 (+- 0.130) |
| N = 8 | Standard (ExEE) | sham | 5.525 (+- 0.150) |
| Von Frey (day 56) N = 8 | ExEE | SNI | 3.45 (+- 0.076) |
| N = 8 | ExEE | Sham | 5.814 (+- 0.188) |
| N = 8 | Standard (ExEE) | SNI | 2.038 (+- 0.056) |
| N = 8 | Standard (ExEE) | sham | 5.725 (+-0.183) |
| Splash test – grooming duration (s) N = 8 | ExEE | SNI | 167.625 (+-10.215) |
| N = 8 | ExEE | Sham | 168 (+-11.458) |
| N = 8 | Standard (ExEE) | SNI | 83.5 (+-8.354) |
| N = 6 | Standard (ExEE) | sham | 114.833 (+-10.995) |
| Tail Suspension Test N = 8 | ExEE | SNI | 39.271 (+- 2.829) |
| N = 8 | ExEE | Sham | 38.958 (+-5.184) |
| N = 8 | Standard (ExEE) | SNI | 60.0 (+-2.78) |
| N = 8 | Standard (ExEE) | sham | 40.521 (+-4.860) |
| Forced Swimming Test N = 8 | ExEE | SNI | 44.375 (+-3.843) |
| N = 8 | ExEE | Sham | 39.618 (+-3.869) |
| N = 8 | Standard (ExEE) | SNI | 60.833 (+-2.151) |
| N = 8 | Standard (ExEE) | sham | 43.715 (+- 1.830) |

| Method | Housing | Condition | Recorded value – mean (SEM) |
| --- | --- | --- | --- |
| cFos/CamKII N = 4 | ExEE | SNI | 38.5 (+-2.6) |
| N = 8 | ExEE | Sham | 34.9 (+-5.4) |
| N = 6 | Standard (ExEE) | SNI | 68.0(+-6.28) |
| N = 4 | Standard (ExEE) | Sham | 42.5 (+-2.9) |
| AP threshold N = 28 | ExEE | SNI | 133.678 (+-16.692) |
| N = 24 | ExEE | Sham | 141.373 (+-20.923) |
| N = 46 | Standard (ExEE) | SNI | 75.436 (+-8.496) |
| N = 24 | Standard (ExEE) | Sham | 130.872 (+-18.715) |
| Input resistance N = 28 | ExEE | SNI | 67.357 (+-3.943) |
| N = 24 | ExEE | Sham | 69.785 (+-4.840) |
| N = 46 | Standard (ExEE) | SNI | 93.817 (+-3.570) |
| N = 24 | Standard (ExEE) | Sham | 73.804 (+-4.236) |
| Firing frequency N = 28 | ExEE | SNI | 16.5 (+-2.449) |
| N = 24 | ExEE | Sham | 14.583 (+-3.3) |
| N = 46 | Standard (ExEE) | SNI | 24.913 (+-1.853) |
| N = 24 | Standard (ExEE) | Sham | 15.167 (+-2.287) |

Figure. 4
